# Early Multidisciplinary Intensive-care Therapy can Improve Outcome of Severe Anti-NMDA-receptor Encephalitis Presenting with Extreme Delta Brush

**DOI:** 10.1515/tnsci-2019-0039

**Published:** 2019-10-02

**Authors:** R. Schneider, M. Brüne, TG. Breuer, C. Börnke, R. Gold, G. Juckel

**Affiliations:** 1 Department of Medicine I, St Josef-Hospital, Ruhr-University Bochum, Bochum, Germany; 2 Department of Psychiatry, Psychotherapy, and Preventive Medicine, Ruhr University Bochum, Bochum, Germany; 3 Department of Medicine I, St Josef-Hospital, Ruhr-University Bochum, Bochum, Germany

**Keywords:** Anti-NMDA receptor encephalitis, Delta Brush, Psychosis, Epileptic seizures - Catatonia, Immunotherapy, Intensive care

## Abstract

Anti-N-methyl-D-aspartate receptor encephalitis (Anti-NMDARE) is a synaptic autoimmune encephalitis syndrome mainly affecting young females. An underlying tumor, most commonly ovarian teratomas in young females, may indicate a paraneoplastic syndrome. Prognostic factors of the clinical course of disease and outcome play a central role in view of early administration of second-line immunotherapy and intensive-care therapy. We report a case of severe Anti-NMDARE associated with unfavorable predictors including an extreme delta brush (EDB) electroencephalographic-pattern and high anti-NMDAR-antibody titers in the cerebral spinal fluid (CSF), which necessitated the admission to an intensive care unit. In spite of the poor prognosis, the patient completely recovered; we attribute this to an early escalation to second-line immunotherapy with rituximab and multidisciplinary intensive-care therapy. The present case underlines the relevance of multidisciplinary management for individuals with Anti-NMDARE.

## Case report

We report the case of a 22-year-old female admitted to a psychiatric ward due to the rapid and acute onset of psychosis, with severe agitation and disorientation. Her symptoms began during a vacation trip. Due to the suspicion of a history of drug use, a drug-induced psychosis was considered as potential differential diagnosis. Severe agitation was first treated with haloperidol and diazepam and later on with quetiapine and lorazepam. One day after hospital admission, the patient developed catatonic stupor, with intermittent episodes of agitation, as well as orolingual-facial dyskinesias followed by abnormal limb movements and epileptic seizures. Brain-MRI demonstrated unspecific bifrontal subcortical T2-hyperintensities without contrast enhancement. The cerebrospinal fluid (CSF) analysis initially showed a moderate lymphocytic pleocytosis (32 lymphocytes/ μl) and a mildly increased protein concentration (460mg/l). Due to physical deterioration with exsiccosis the patient was transferred to an intensive care unit.

As a consequence of liquor pleocytosis antiviral and antibacterial medication was initiated. Normal pathogen-findings and verification of NMDA receptor antibodies in CSF (1:32) and serum (1:100) lead to an intravenous prednisolone administration as first-line anti-inflammatory therapy. However, the patient developed seizures with increasing frequency which culminated in status epilepticus. The patient was subsequently switched from levetiracetam to lamotrigine.

**Figure 1:**
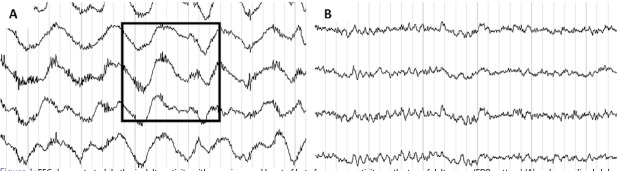
EEG demonstrated rhythmic delta activity with superimposed burst of beta frequency activity on the top of delta wave (EDB-pattern) (A) and normalized alpha frequency activity during follow-up EEG after initiation of immunotherapy (B).

Periods of reduced levels of consciousness with diminished response to painful stimuli occurred independently of epileptic seizures. Electroencephalography (EEG) showed rhythmic delta activity with superimposed burst of beta frequency activity on the top of delta wave (Figure 1 A), known as “extreme delta brush” (EDB) pattern, which is considered typical for anti-NMDA encephalitis [1].

Gynecological examination with Tumor-screening including teratoma was inconspicuous. Thus, alternating immunoadsorption and plasma exchange was started. Episodes of agitation with self-removing of the naso-gastric tube resulted in deficient liquid and food intake. A percutaneous endoscopic gastrostomy (PEG) tube, which was tolerated much better, was inserted to ensure alimentation. Afterward a gradual improvement regarding the intermittent reduced level of consciousness, agitation and the occurrence of epileptic seizures was observed, while cognitive deficits persisted. Intensive physical and logopedic therapy improved mobilization and self-employed ingestion. After completion of therapeutic plasma exchange and PEG placement we started second-line immunotherapy with 500mg rituximab. With respect to complete remission of psychotic symptoms and epileptic seizures anti-psychotic and anti-epileptic medication was reduced. Furthermore, cognition appeared normal. Normal alpha frequency activity replaced pathological EEG findings with EDB. Finally, the patient was transferred to a rehabilitation center two months after onset of acute psychosis.

After the renewed increase of CD19 B-cell count, immunotherapy with rituximab was continued at 6 and 16 months after the first rituximab administration. To date the patient returned to a normal state of health without any restrictions and was able to complete her university education successfully.

## Discussion

Anti-NMDARE is the most frequent synaptic autoimmune encephalitis syndrome. Children and young, mainly female adults are primarily affected. The disease is associated with IgG antibodies to the GluN1 subunit of the NMDA receptor [2].

With regard to prognosis, there are several conditions leading to a more favorable course of disease and outcome. Predictors of good outcome are early treatment and no admission to an intensive care unit [3]. Furthermore, Glasgow Coma Scale score ≤8 at admission, number of complications, and admission to an intensive care unit were described as predictors of death by Chi et al [4]. The concurrence with an associated ovarian tumor, influences the response to first-line immunotherapy and necessity of additional therapy [2].

Higher CSF titers of Anti-NMDAR-antibodies predict an impaired clinical outcome [5]. Furthermore, the EDB is associated with the occurrence of electroclinical seizures and a more prolonged illness [1].

We report a case of a severe course of Anti-NMDARE fulfilling conditions indicative of a prolonged disease and poor outcome. Even so an early multidisciplinary intensive-care therapy, included psychiatrist for anti-psychotic treatment, neurologist for anti-epileptic therapy, internal specialist for nutrition management, physical and ergotherapeutical therapists for remobilization and cognition training as well as administration of immunotherapy led to full recovery of health.

The clinical course of disease in this case was typically characterized by initial psychotic symptoms followed by catatonia, abnormal movements and seizures complicated by altered mental status [2]. Progressive frequency of seizures required escalation of antiepileptic medication and intensive care monitoring. Adults with Anti-NMDAR encephalitis present epileptic seizures in 76% of cases [6]. Apart from typically visible diffuse background slowing or focal slow waves mostly in frontotemporal regions, there is a specific electrographic pattern characterized by rhythmic delta activity with overlaid bursts of rhythmic beta frequency activity on delta waves, named “extreme delta brush” (EDB). EDB occurs in up to 30% of patients with NMDARE [1]. Schmitt et al. suggest that EDB is indicative of severity and perhaps predictive of poor outcome [1]. These findings were confirmed by Steriade et al. who described extreme delta, with or without brushes, as a surrogate marker of disease activity in NMDARE refractory to first line immunotherapy [7]. The EDB pattern could also be demonstrated in our case (Figure 1A) and resolution of EDB occurred after initialization of second-line immunotherapy and clinical improvement (Figure 1B)

In the present case high CSF and serum titers of Anti-NMDAR-antibodies suggested a poorer outcome [5]. High antibody titers are associated with the presence of a teratoma [5], thus gynecological examination played a central role in this case.

An extensive tumor-screening included gynecological examination, Fluorine-18 fluorodeoxyglucose positron emission tomography scan of the thorax and abdomen, blood smear and laboratory-chemical examinations of tumor-markers. Tumor-screening provided no indication for a paraneoplastic cause. The occurrence of teratoma in female patients older than 12 years amounts to 52% [3]. Albeit the final outcome and substantial improvement are the same in patients with or without tumor, patients without tumor do not improve after first-line immunotherapy as well as patients with tumor [2]. This entails that additional second-line immunotherapy is required resulting in prolonged duration of therapy [8]. Additional episodes of unconsciousness and confusion can be longer or worse in patients without tumors [2]. In our patient clinical improvement after methylprednisolone and plasma exchange was deficient. Conclusively, we initiated an early second-line immunotherapy with Rituximab 4 weeks after clinical onset of disease.

Nutrition management was another complication during the course of severe illness with progressive frequency of epileptic seizures and increased aspiration-risk. Furthermore, alternating periods of agitation lead to dislocation of the naso-gastric tube. Conclusively, nutrition-management poses a challenge owing to long disease duration in patients with Anti-NMDARE. Nutrition is an important consideration in intensive care management in patients with high aspiration risk in consequence of seizures and intolerance for a naso-gastric tube resulting from agitation. We decided for an early enteral nutrition based on consensus of nutrition of critically ill patients [9]. With administration of a transient naso-gastric tube switching to a PEG tube

we improved compliance. Additionally early mobilization and early deglutition-training based on logopedics field of expertise were performed after clinically stabilization to improve clinical and functional recovery [10].

In summary the present case illustrates a severe course of Anti-NMDARE with conditions indicative of a prolonged disease and poor outcome like absence of tumor-association, high CSF titers of Anti-NMDAR-antibodies, presence of EDB pattern in EEG, need of admission to an intensive care unit and challenging nutrition-management. Nevertheless, multidisciplinary intensive-care therapy led to a positive course of disease with full recovery. Early diagnosis and early implementation of primary immunotherapy are mandatory as described in literature [3]. In the present case first line therapy resulted in inadequate improvement of cognition. Additionally, unfavorable predictors like EDB-pattern and high CSF-titers led to an early administration of second-line immunotherapy with Rituximab. Even though an admission to ICU is considered as predictor of poor outcome and death [3, 4], in the present case multidisciplinary intensive-care therapy was required for intensive-care challenges like complex nutrition-management, early mobilization and deglutition-training. Thus, we agree with De Montmollin et al. who described prognosis of anti-NMDARE in adults requiring intensive care as good, when immunotherapy is initiated early and argued in favor of prompt diagnosis and early aggressive treatment [11].

In conclusion adults with anti-NMDARE and unfavorable predictors like EDB-pattern and high Anti-NMDAR-antibody- CSF-titers are not necessarily linked to poor prognosis. Rapid implementation of second-line immunotherapy should be performed. An accompanying multidisciplinary intensive-care therapy may result in an effective clinical and functional recovery.
